# Stress hyperglycemia is associated with poor outcome in critically ill patients with pulmonary hypertension

**DOI:** 10.3389/fendo.2024.1302537

**Published:** 2024-02-23

**Authors:** Chuyan Long, Weiguo Fan, Yang Liu, Kui Hong

**Affiliations:** ^1^ Department of Cardiology, The Second Affiliated Hospital of Nanchang University, Nanchang, Jiangxi, China; ^2^ Department of Genetic Medicine, The Second Affiliated Hospital of Nanchang University, Nanchang, Jiangxi, China; ^3^ Jiangxi Key Laboratory of Molecular Medicine, Second Affiliated Hospital of Nanchang University, Nanchang, Jiangxi, China

**Keywords:** pulmonary hypertension, stress, hyperglycemia, intensive care unit, mortality

## Abstract

**Background and objective:**

Stress hyperglycemia is common in critically ill patients and is associated with poor prognosis. Whether this association exists in pulmonary hypertension (PH) patients is unknown. The present cohort study investigated the association of stress hyperglycemia with 90-day all-cause mortality in intensive care unit (ICU) patients with PH.

**Methods:**

Data of the study population were extracted from the Medical Information Mart for Intensive Care IV (MIMIC-IV) database. A new index, the ratio of admission glucose to HbA1c (GAR), was used to evaluate stress hyperglycemia. The study population was divided into groups according to GAR quartiles (Q1-Q4). The outcome of interest was all-cause mortality within 90 days, which was considered a short-term prognosis.

**Result:**

A total of 53,569 patients were screened. Ultimately, 414 PH patients were enrolled; 44.2% were male, and 23.2% were admitted to the cardiac ICU. As the GAR increased from Q2 to Q4, the groups had lower creatinine levels, longer ICU stays, and a higher proportion of renal disease. After adjusting for confounding factors such as demographics, vital signs, and comorbidities, an elevated GAR was associated with an increased risk of 90-day mortality.

**Conclusion:**

Stress hyperglycemia assessed by the GAR was associated with increased 90-day mortality in ICU patients with PH.

## 1 Introduction

Pulmonary hypertension (PH) is a complicated disease characterized by occlusive pulmonary arterioles and progressive increases in pulmonary artery pressure and vascular resistance, which result from multiple factors (1, 2) and significantly shorten the life expectancy of patients. The mortality rate among PH patients ranges from 38% to 63% (3), even if they receive regular treatment. Patients with PH often require ICU admission due to severe end-stage symptoms, such as worsened cardiac failure and hypoxemia, and have higher mortality rates than patients without PH (4, 5). Sztrymf et al. reported that the mortality rate is as high as 41% in the PH population in the ICU. No significant differences were found between survivors and nonsurvivors in baseline characteristics and hemodynamic data collected on admission (6). Identifying the factors that impact the prognosis of PH patients in the ICU holds significant clinical value.

Thanks to advances in medicine, the prognostic value of many clinical indexes has been well established in patients with PH. These predictors can be categorized as invasive or noninvasive indexes. Right atrial pressure (RAP), the cardiac index (CI) and mixed venous oxygen saturation (SvO_2_) are the most robust invasive indicators of prognosis (7–10). The World Health Organization functional class (WHO-FC) (11), 6-minute walk distance (6MWD) (12, 13) and N-terminal pro-B-type natriuretic peptide (NT-proBNP) (7, 14) level are recognized as valuable noninvasive prognostic factors for PH patients. These factors have some limitations; for example, elevated NT-proBNP levels can be seen in almost any heart disease patient and tend to show high variability (15). In addition, wide variation in clinicians’ assessments of WHO-FC in patients with PH exists (16). It is difficult for critically ill patients in the ICU to perform the 6MWD test. The data from right heart catheterization are very predictable, but the invasive operation limits its wide clinical application.

Stress-induced hyperglycemia (SIH) is a temporary condition in hospitalized patients with acute illnesses that resolves independently after the illness subsides (17). SIH is common in critically ill patients, even in those without diabetes (18, 19), and growing evidence suggests that hyperglycemia is linked to higher mortality (20–22). Under different stress situations, the mortality rate of patients with hyperglycemia ranges from 16% to 40%, which is significantly higher than that of patients with lower blood glucose levels (1.7-11%) (23–26).

There is no evidence to suggest that SIH is associated with the prognosis of PH patients. Glycated hemoglobin (HbA1c) represents the standard average glucose level over the previous three months. The glucose-to-HbA1c ratio (GAR) indicates the extent of the increase in a patient’s plasma glucose level over the background levels, representing the intensity of SIH. We used the GAR (27) to investigate the impact of SIH on PH patient outcomes in the search for a new predictive index to reduce the short-term mortality of PH patients in the ICU.

## 2 Methods

### 2.1 Study population

**Figure 1:**
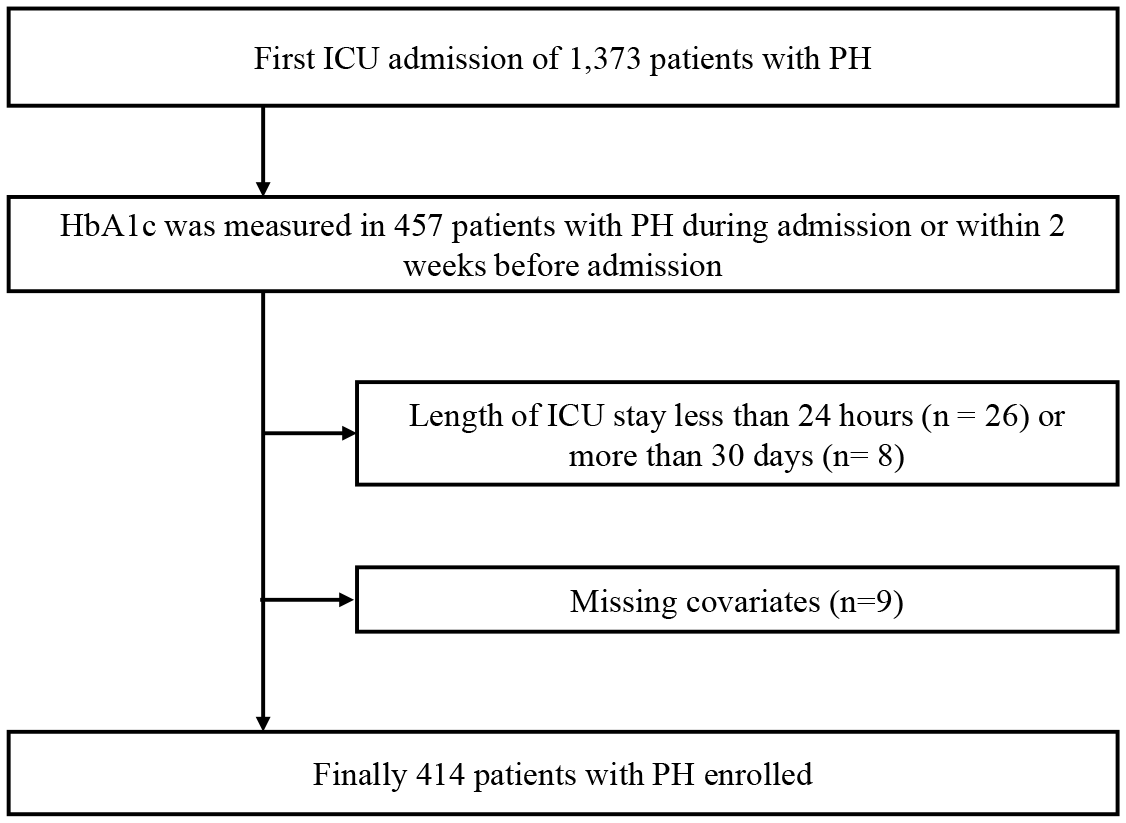
Flow diagram for patient screening. PH, pulmonary hypertension.

The Medical Information Mart for Intensive Care IV (MIMIC-IV) database version 2.0 is a freely available open critical care database. In this study, the critical data included detailed demographic characteristics, clinical features, diagnosis, treatment, and other information from 53,569 ICU patients admitted to the Beth Israel Deaconess Medical Center in Boston, Massachusetts, between 2008 and October 2019. An approved researcher, Yang Liu (certification number: 55,302,712), extracted the study data; the code for data query and extraction is available from the MIMIC Code Repository (https://github.com/MIT-LCP/mimic-code). In the database, disease diagnosis was mainly based on the International Classification of Diseases, Ninth and Tenth Revision (ICD-9 and ICD-10) codes documented by hospital staff. We identified 1,373 patients with a PH diagnosis, defined by ICD-9 code 4160 and ICD-10 codes I270, I272, I2720, I2722-I2724, and I2729. Patients without HbA1c data, those with a length of ICU stay less than 24 hours or more than 30 days, and those with missing covariates were excluded (**Figure 1**).

### 2.2 Measurement of the GAR

We extracted the first measurement values of blood glucose during the ICU stay as admission glucose levels. To ensure that the HbA1c level reflected the average blood glucose level from 8 to 12 weeks before ICU admission, only HbA1c values from 2 weeks before to 3 days after admission were gathered. The GAR was calculated to evaluate stress hyperglycemia, and the study population was divided into groups according to GAR quartile(Q1: GAR < 20.1; Q2: 20.1 ≤ GAR<22.6; Q3: 22.6 ≤ GAR < 25.0; Q4: GAR ≥ 25.0).

### 2.3 Study outcomes

The ICU admission date was defined as the index date, and the study outcome of interest was all-cause mortality within 90 days. The date of death was derived from hospital records and state records, and the latter were matched using a custom rule-based linkage algorithm based on name, date of birth, and social security number. Due to the lack of ICD codes for death records, we did not analyze specific causes of death.

### 2.4 Covariates

The covariates included demographics (age, sex, and race), the Simplified Physiological Score II (SPAS II), vital signs (mean arterial pressure, respiratory rate, pulse oxygen saturation (SPO_2_), and urine output) and comorbidities (heart failure (HF), cerebrovascular disease and renal dysfunction).

### 2.5 Statistical analyses

Continuous variables are expressed as the mean ± standard deviation (SD) or median and interquartile range (IQR), whose values at baseline were compared between groups by one-way analysis of variance or the Kruskal−Wallis test, respectively. Categorical variables are expressed as numbers and percentages (%), and differences were examined by Fisher’s exact test. Kaplan−Meier curves and log-rank tests were used to compare the difference in 90-day mortality among the four GAR quartile groups. Cox proportional regression was used to determine the association between the GAR and 90-day mortality, and the hazard ratio (HR) and 95% confidence interval (CI) are reported. Age, sex, race, the SPAS II, mean arterial pressure, respiratory rate, SPO_2_, urine output and comorbidities (HF, cerebrovascular disease and renal dysfunction) were adjusted for. Moreover, a four-knot (P25, P50, P75, P95) restricted cubic spline (RCS) was used to show a possible nonlinear association between the GAR as a continuous variable and 90-day mortality. In addition, subgroup analyses were performed, and interaction effects were calculated based on age (< or ≥ 70 years), sex (male or female), the presence or absence of HF and HbA1c level (< or ≥ 6%).

All statistical analyses were performed by Stata version 17 (Stata Corp), and a *P* value less than 0.05 was considered statistically significant.

## 3 Results

### 3.1 Baseline characteristics of PH patients based on the GAR

The 414 patients diagnosed with PH who were included were divided into four quartile groups (Q1-Q4) according to the GAR, and the baseline characteristics were compared. There were no differences in age, sex, race, blood pressure, heart rate, respiratory rate, urine output, or the diagnosis of HF or cerebrovascular disease among the four groups (**Table 1**). Patients with higher GAR values had higher admission blood glucose levels (P < 0.001), while the HbA1c level decreased in the Q1 to Q3 (GAR < 25.0) groups and then increased in the Q4 group (GAR ≥ 25.0) (P < 0.001). There was a trend towards increased creatinine levels (P < 0.001), longer ICU stays (P = 0.003), and higher proportions of renal disease (P = 0.011) from the Q2 to Q4 groups. However, compared to the Q2 group, the Q1 group had a higher SAPS II and serum creatinine level, lower SPO_2_, and a longer ICU stay, while the GAR was lowest.

**Table 1:** Baseline characteristics according to GAR (glucose-HbA1c ratio) quartile.

Characteristics	Overall (n=414)	Q1 (< 20.1) (n=104)	Q2 (20.1-22.6) (n=105)	Q3 (22.6-25.0) (n=102)	Q4 (≥25.0) (n=103)	P value
Age, years, median (IQR)	71 (62-79)	68 (59-80)	73 (67-78)	68 (61-77)	72 (63-80)	0.564
Race, white, n (%)	263 (63.5)	59 (56.7)	76 (72.4)	65 (63.7)	63 (61.2)	0.109
Cardiac ICU, n (%)	96 (23.2)	33 (31.7)	17 (16.2)	19 (18.6)	27 (26.2)	0.031
SAPS II, median (IQR)	36 (29-44)	34 (28-41)	34 (29-41)	36 (28-44)	40 (31-49)	0.014
Sex, male, n (%)	183 (44.2)	61 (58.7)	59 (56.2)	55 (53.9)	56 (54.4)	0.901
Systolic blood pressure, mmHg, median (IQR)	112 (106-120)	113 (104-126)	112 (107-119)	111 (106-117)	110 (105-120)	0.731
Diastolic blood pressure, mmHg, median (IQR)	60 (54-66)	61 (54-70)	60 (55-65)	58 (52-64)	60 (54-70)	0.100
Mean arterial pressure, mmHg, median (IQR)	76 (71-83)	76 (71-86)	78 (72-82)	75 (69-80)	76 (71-83)	0.256
Heart rate, beats/min, median (IQR)	80 (74-90)	79 (73-90)	80 (73-88)	80 (73-90)	83 (75-93)	0.165
Respiratory rate, beats/min, median (IQR)	19 (17-22)	19 (16-22)	18 (17-20)	19 (17-22)	19 (17-23)	0.150
SPO_2_, %, median (IQR)	97 (96-98)	97 (95-98)	97 (96-98)	98 (96-98)	97 (96-98)	0.039
Urine output, ml/day, median (IQR)	1650 (1050-2330)	1613 (1026-2223)	1730 (1187-2355)	1669 (1125-2195)	1548 (845-2445)	0.890
Length of ICU stay, day, median (IQR)	3.0 (1.5-5.2)	3.2 (2.0-5.1)	2.1 (1.3-4.2)	3.0 (1.5-4.9)	3.3 (2.0-6.9)	0.003
Admission blood glucose, mg/dL, median (IQR)	130 (120-149)	119 (107-134)	124 (118-134)	130 (123-142)	155 (140-206)	< 0.001
HbA1c, %, median (IQR)	5.8 (5.4-6.4)	6.4 (5.8-7.8)	5.8 (5.5-6.2)	5.4 (5.2-6.0)	5.6 (5.1-6.1)	< 0.001
Creatinine, mg/dL, median (IQR)	1.0 (0.8-1.4)	1.0 (0.8-1.4)	0.9 (0.7-1.3)	0.9 (0.7-1.3)	1.1 (0.9-1.7)	< 0.001
Heart failure, n (%)	262 (63.3)	69 (66.4)	61 (58.1)	61 (59.8)	71 (68.9)	0.310
Cerebrovascular disease, n (%)	69 (16.7)	22 (21.2)	15 (14.3)	16 (15.7)	16 (15.5)	0.552
Renal disease, n (%)	114 (27.5)	35 (33.7)	20 (19.2)	22 (21.4)	37 (35.9)	0.011

IQR, interquartile range.

### 3.2 Association between the GAR quartile and 90-day mortality

**Figure 2:**
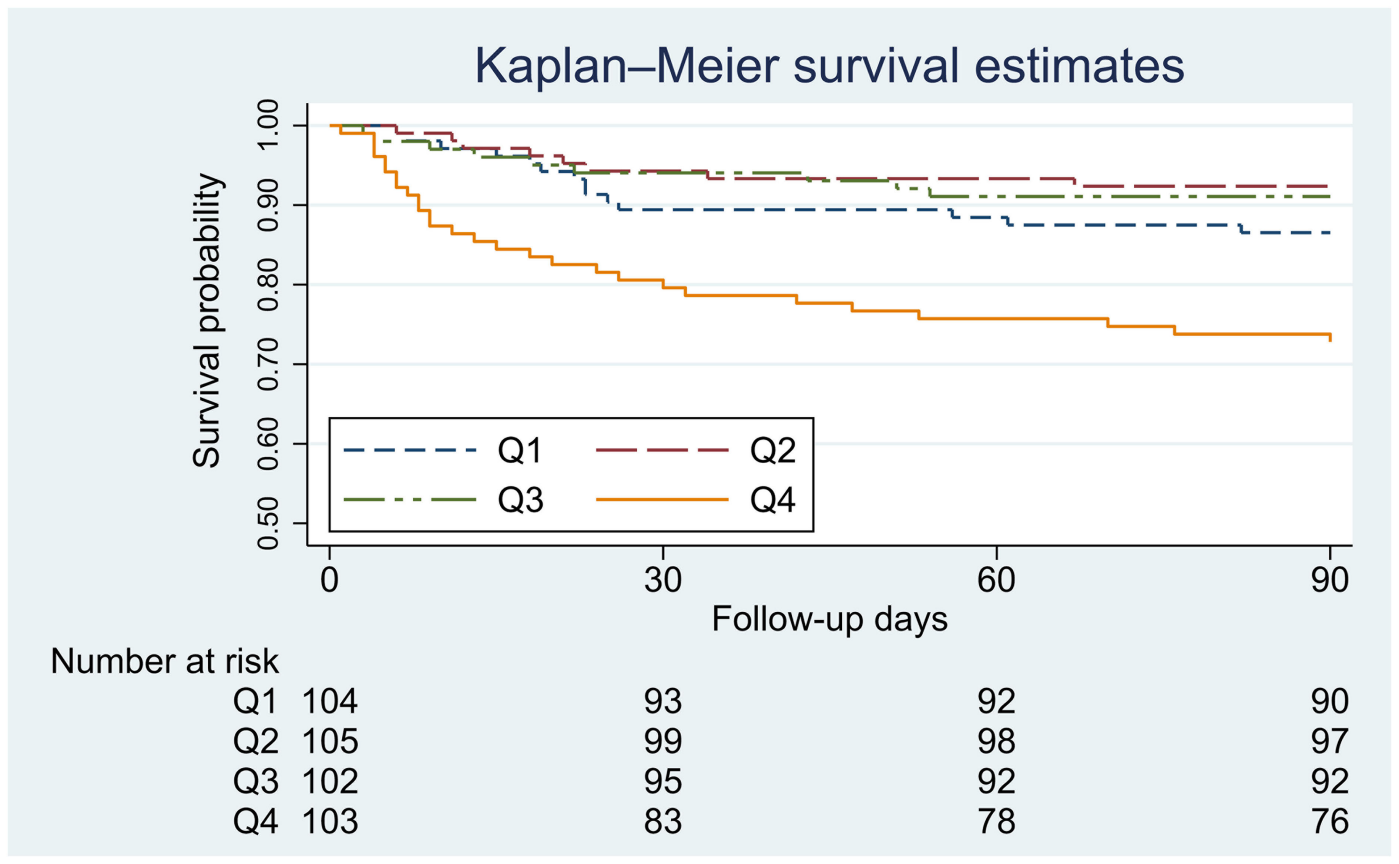
Kaplan−Meier survival curves for GAR and 90-day mortality. Q1: GAR < 20.1; Q2: 20.1 ≤ GAR<22.6; Q3: 22.6 < GAR ≤ 25.0; Q4: GAR > 25.0.

The primary outcome of the present study was all-cause mortality within 90 days following initial admission to the ICU. Since the mortality of patients in the Q2 group was the lowest, it was used as a reference in the following analysis (**Table 2**). The 90-day mortality was 14.3% (59/414) in all patients with PH. The subjects in the Q4 group (GAR ≥ 25.0) showed the highest 90-day mortality (27.2% *vs*. 7.6%, P < 0.001). The subjects in the Q1 group (GAR < 20.1) had a slightly but not significantly higher 90-day mortality rate than those in the Q2 group (13.5% *vs*. 7.6%) (P = 0.169). There was no difference between the Q3 and Q2 groups (8.8% *vs*. 7.6%, P = 0.752). Kaplan−Meier survival curves showed that PH patients in the Q4 group had a lower survival probability than those in the other three quartile groups (log-rank P < 0.001) (**Figure 2**).

**Table 2:** The association between quartile of GAR and 90-day mortality by Cox proportional-hazard regression.

Variable	Q1, N=104 (< 20.1)	Q2, N=105 (20.1 to < 22.6)	Q3, N=102 (22.6 to < 25.0)	Q4, N=103 (≥25.0)
90-day mortality (%)	14 (13.5)	8 (7.6)	9 (8.8)	28 (27.2)
Crude, HR (95% CI)	1.80 (0.76-4.30)	Reference	1.17 (0.45-3.03)	4.07 (1.86-8.93)
Model 1, HR (95% CI)	1.86 (0.77-4.49)	Reference	1.24 (0.48-3.24)	3.98 (1.81-8.77)
Model 2, HR (95% CI)	1.45 (0.55-3.78)	Reference	1.02 (0.38-2.76)	2.73 (1.21-6.17)

HR, hazard ratio; CI, confidence interval.

Model 1: adjusted for age, sex and race.

Model 2: adjusted for age, sex, race, category of ICU, SAPS II, heart failure, cerebrovascular disease, renal dysfunction, mean arterial pressure, respiratory rate, SPO_2_, and urine output.

**Figure 3:**
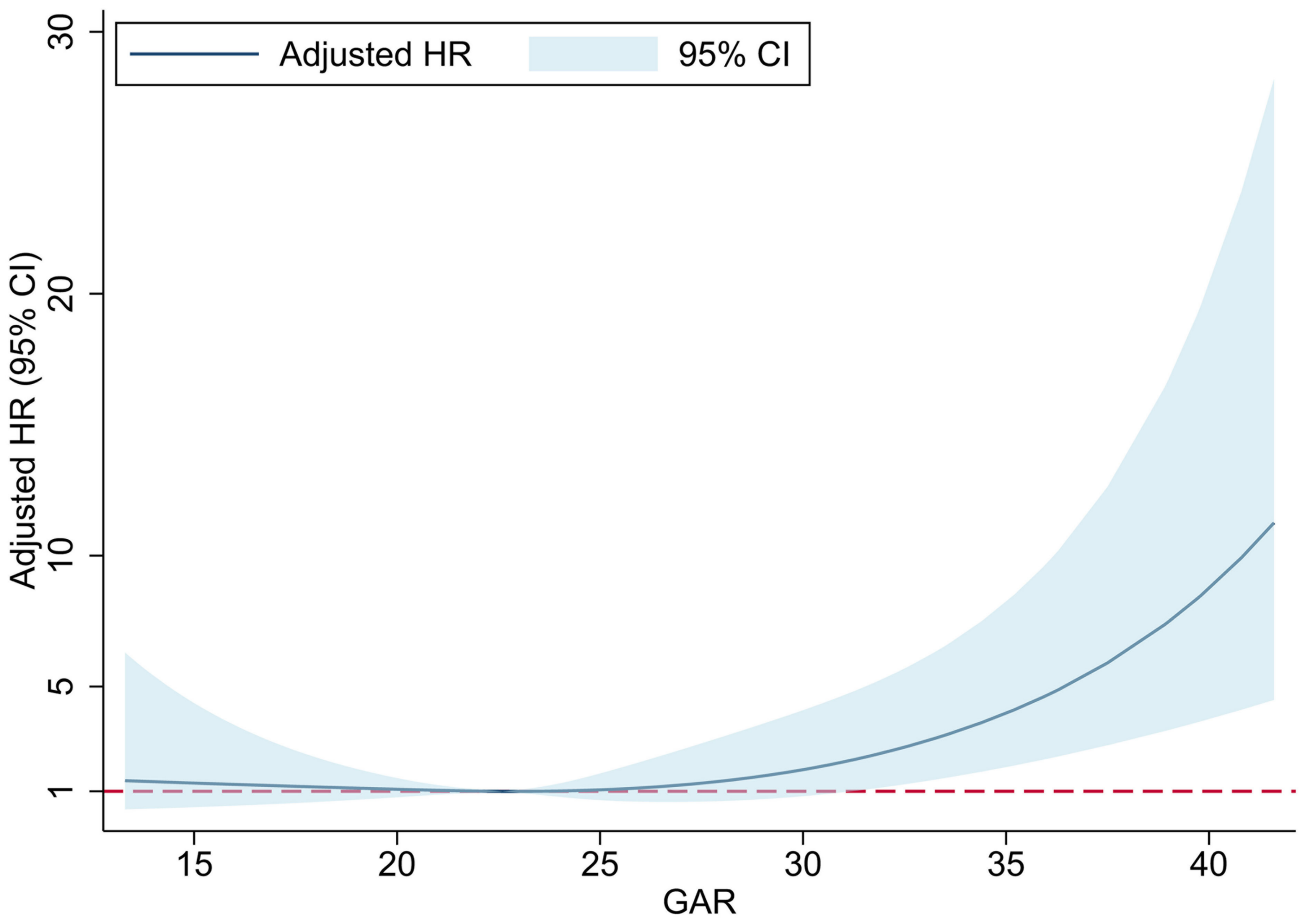
The association between GAR and 90-day mortality. GAR, The glucose-to-HbA1c ratio; HR, hazard ratio; CI, confidence interval.

In the unadjusted Cox proportional hazard model, the Q4 group had a higher risk of 90-day mortality than the Q2 group (HR 4.07, 95% CI: 1.86-8.93, P < 0.001). There were no differences among the Q1, Q2, and Q3 groups. We built two adjusted models to reduce the impact of confounding factors (**Table 2**). Model 1 was adjusted for demographics (age, sex and race), and the results were consistent with those of the crude model. Model 2 was fully adjusted for demographics, category of ICU, the SAPS II, HF, cerebrovascular disease, renal dysfunction, mean arterial pressure, respiratory rate, SPO_2_, and urine output. The association between a higher GAR and mortality was still significant (HR 2.73, 95% CI: 1.21-6.17, P = 0.016). The association between the GAR as a continuous variable and 90-day mortality is shown in **Figure 3**. Finally, an insignificant nonlinear correlation was found.

### 3.3 Subgroup analyses

In the subgroup analyses, we divided the enrolled PH patients into two groups based on age (< or ≥ 70 years), sex (male or female), the presence or absence of HF, and HbA1c level (< or ≥ 6%). A multivariate Cox regression model was used for the analysis. In addition to the above four parameters, confounding factors such as demographics, category of ICU, SAPS II, cerebrovascular disease, renal dysfunction, mean arterial pressure, respiratory rate, SPO_2_, and urine output were also adjusted in the model. The results showed that the HR of 90-day mortality in the Q4 group (GAR ≥ 25.0) was higher than that in the Q1-Q3 groups. The HR was similar between patients with and without HF. In addition, elderly patients (≥ 70 years: HR 4.22, 95% CI: 1.98-8.99), female patients (HR 4.36, 95% CI: 1.62-11.72), and patients with an HbA1c level ≥ 6% (HR 5.28, 95% CI: 2.07-13.46) had a higher HR of 90-day mortality; however, there were no significant differences among the GAR quartile groups (**Table 3**). We conclude that the association between an elevated GAR and mortality was consistent in different subsets of PH patients. It would be a good idea to conduct RCTs with larger populations to confirm this hypothesis.

**Table 3:** Subgroup analyses of GAR (Q4 *vs*. Q1-Q3) and 90-day mortality.

Subgroups	N	Adjusted HR (95% CI)	P for interaction
Age, years			
< 70	194	1.31 (0.39-4.33)	0.117
≥ 70	220	4.22 (1.98-8.99)	
Sex			
Male	231	2.08 (0.92-4.71)	0.373
Female	183	4.36 (1.62-11.72)	
Heart failure			
No	152	2.74 (0.79-9.50)	0.541
Yes	262	2.98 (1.49-5.93)	
HbA1c, %			
< 6	244	3.64 (1.36-9.75)	0.335
≥ 6	170	5.28 (2.07-13.46)	

HR, hazard ratio; CI, confidence interval.

## 4 Discussion

A total of 414 PH patients from the MIMIC Critical Care Data Center were retrospectively screened. There were no significant differences in age, sex, or race among the patients with different GAR values at baseline. The length of ICU stay, creatinine level, and proportion of patients with kidney disease gradually increased as the GAR increased within a certain range (Q2-Q4: GAR ≥ 20.1). This indicated that SIH might be related to the severity of PH. The analysis also found that an elevated GAR was associated with poorer 90-day survival in PH patients in the ICU. In addition to demographic characteristics such as age, sex, race, and ICU category, our analyses were adjusted for underlying diseases that may affect a patient’s short-term prognosis, including HF, cerebrovascular disease, and renal dysfunction. Furthermore, indexes reflecting the severity of disease in the ICU, such as the SAPS II, SPO_2_, mean arterial pressure, respiratory rate and urine output, were adjusted for. The present results suggest a correlation between the severity of disease and the increase in blood glucose when PH patients experience stress. After adjusting for the above confounders, the association between an elevated GAR and increased risk of 90-day mortality remained. When patients were divided into two groups according to age, sex, complications of HF, or HbA1c level, an elevated GAR was still associated with mortality.

Some studies suggest that blood glucose levels should be considered a new vital sign indicative of prognosis during hospitalization (28–30). The GAR can be considered a measurement of increases in plasma glucose during stress. Su Y.W. et al, reported that the GAR can independently predict 90-day mortality and ICU admission for patients with extreme hyperglycemia during acute illness (27). The analysis of this study showed that the GAR was a useful predictor of poor prognosis in patients suffering acute stress. Our results suggest that the association between the GAR and poor outcomes also exists in PH patients in the ICU.

SIH is an evolutionarily preserved response to severe stress (31), which is a temporary state of insulin resistance and deficiency (32). It has been argued that appropriate SIH is protective and serves as an adaptive response to threats (31, 33). In animal models of hemorrhagic shock, the administration of a hypertonic glucose solution improved survival and increased cardiac output and blood pressure (34). In humans, some data showed that plasma concentrations of epinephrine increased fifty-fold and norepinephrine levels increased tenfold (35) in patients with shock. Experimental data show that abnormal epinephrine increases during stress states can stimulate glucagon secretion and inhibit insulin release by pancreatic β-cells (36). Adrenal cortisol output can increase up to tenfold with severe stress (37), promote hepatic gluconeogenesis and glycogenolysis and increase blood glucose (38, 39). In addition, acute stress increases the secretion of proinflammatory cytokines, including tumor necrosis factor alpha (TNF-α), interleukin-1 (IL-1) and interleukin-6 (IL-6) (40–42). These inflammatory factors increase insulin resistance by interfering with the insulin signaling pathway, further increasing blood glucose.

Disorders of glucose metabolism characterized by poor glycemic control and insulin resistance are common in PH patients. The proportion of PH patients who have diabetes mellitus exceeds 20% (43–45). Pulmonary artery smooth muscle cells (PASMCs) stimulated by hyperglycemia are more oxidatively stressed and generate higher levels of superoxide anion (O_2_^·−^) (46), which is the primary form of the mitochondrial free radical leading to vascular cell damage (47). Therefore, excessively high blood glucose may aggravate oxidative stress and promote vascular damage in PH patients.

Our study provides the first evidence for the prognostic value of the GAR in specific populations. These results suggest that stress hyperglycemia is a predictor of poor short-term outcomes for PH patients in the ICU. Moreover, the patients in the Q1 group appeared to have a higher 90-day mortality rate than those in the Q2 group, but the difference was not significant. Some studies focused on emergency and ICU populations found that relative hypoglycemia was associated with an increased risk of mortality and ventricular arrhythmia (48, 49). Similarly, the higher mortality rate in our study’s low GAR group (GAR < 20.1) may indicate poorer physiological conditions of these patients at admission. We hypothesize that a low GAR value also impacts the prognosis of PH patients in the ICU. Of course, the lack of a significant difference necessitates a larger sample to draw firm conclusions.

We conclude that the GAR has an effect on the short-term prognosis of PH patients in the ICU, providing an indicator for predicting the prognosis of critically ill patients with PH. Our findings remind clinicians of the importance of blood glucose management.

## 5 Limitations

The population of PH patients included in this study was small, and data on different PH subtypes could not be extracted from the MIMIC-IV database. Additionally, in this retrospective study, few data were available on glycated blood proteins. The above limitations could result in some bias in the results. Furthermore, the prognostic value of the GAR in PH patients was assessed within a specific population in the ICU. Therefore, prospective studies based on larger cohorts are still needed to validate the association between the GAR and outcomes in patients with PH in mild or non-ICU populations.

## 6 Conclusion

Stress hyperglycemia assessed by the GAR was associated with increased 90-day mortality in ICU patients with PH.

## Data availability statement

The original contributions presented in the study are included in the article/supplementary material. Further inquiries can be directed to the corresponding author.

## Ethics statement

The studies involving humans were approved by the Institutional Review Boards of Beth Israel Deaconess Medical Center; Institutional Review Boards of Massachusetts Institute of Technology. The studies were conducted in accordance with the local legislation and institutional requirements. The ethics committee/institutional review board waived the requirement of written informed consent for participation from the participants or the participants’ legal guardians/next of kin due to the use of anonymous clinical data.

## Author contributions

CL: Formal Analysis, Writing – original draft, Writing – review & editing. WF: Formal analysis, Writing – original draft, Data curation. YL: Validation, Writing – review & editing. KH: Funding acquisition, Supervision, Writing – review & editing.

## References

[1] Hansmann G. Pulmonary hypertension in infants, children, and young adults. J Am Coll Cardiol (2017) 69:2551–69. doi: 10.1016/j.jacc.2017.03.575 28521893

[2] Southgate L MaChado RD Graf S Morrell NW. Molecular genetic framework underlying pulmonary arterial hypertension. Nat Rev Cardiol (2020) 17:85–95. doi: 10.1038/s41569-019-0242-x 31406341

[3] Leuchte HH Ten Freyhaus H Gall H Halank M Hoeper MM Kaemmerer H. Risk stratification strategy and assessment of disease progression in patients with pulmonary arterial hypertension: Updated Recommendations from the Cologne Consensus Conference 2018. Int J Cardiol (2018) 272S:20–9. doi: 10.1016/j.ijcard.2018.08.084 30266353

[4] Frank DB Crystal MA Morales DL Gerald K Hanna BD Mallory GB Jr.. Trends in pediatric pulmonary hypertension-related hospitalizations in the United States from 2000-2009. Pulm Circ (2015) 5:339–48. doi: 10.1086/681226 PMC4449246 26064460

[5] Maxwell BG Nies MK Ajuba-Iwuji CC Coulson JD Romer LH. Trends in hospitalization for pediatric pulmonary hypertension. Pediatrics (2015) 136:241–50. doi: 10.1542/peds.2014-3834 26148956

[6] Sztrymf B Souza R Bertoletti L Jais X Sitbon O Price LC. Prognostic factors of acute heart failure in patients with pulmonary arterial hypertension. Eur Respir J (2010) 35:1286–93. doi: 10.1183/09031936.00070209 19897557

[7] Nickel N Golpon H Greer M Knudsen L Olsson K Westerkamp V. The prognostic impact of follow-up assessments in patients with idiopathic pulmonary arterial hypertension. Eur Respir J (2012) 39:589–96. doi: 10.1183/09031936.00092311 21885392

[8] Benza RL Miller DP Gomberg-Maitland M Frantz RP Foreman AJ Coffey CS. Predicting survival in pulmonary arterial hypertension: insights from the Registry to Evaluate Early and Long-Term Pulmonary Arterial Hypertension Disease Management (REVEAL). Circulation (2010) 122:164–72. doi: 10.1161/CIRCULATIONAHA.109.898122 20585012

[9] McLaughlin VV Sitbon O Badesch DB Barst RJ Black C Galie N. Survival with first-line bosentan in patients with primary pulmonary hypertension. Eur Respir J (2005) 25:244–9. doi: 10.1183/09031936.05.00054804 15684287

[10] Sitbon O McLaughlin VV Badesch DB Barst RJ Black C Galie N. Survival in patients with class III idiopathic pulmonary arterial hypertension treated with first line oral bosentan compared with an historical cohort of patients started on intravenous epoprostenol. Thorax (2005) 60:1025–30. doi: 10.1136/thx.2005.040618 PMC1747276 16055621

[11] Barst RJ Chung L Zamanian RT Turner M McGoon MD. Functional class improvement and 3-year survival outcomes in patients with pulmonary arterial hypertension in the REVEAL Registry. Chest (2013) 144:160–8. doi: 10.1378/chest.12-2417 23429998

[12] Fritz JS Blair C Oudiz RJ Dufton C Olschewski H DeSpain D. Baseline and follow-up 6-min walk distance and brain natriuretic peptide predict 2-year mortality in pulmonary arterial hypertension. Chest (2013) 143:315–23. doi: 10.1378/chest.12-0270 PMC4694187 22814814

[13] Gaine S Simonneau G. The need to move from 6-minute walk distance to outcome trials in pulmonary arterial hypertension. Eur Respir Rev (2013) 22:487–94. doi: 10.1183/09059180.00006213 PMC9639189 24293464

[14] Williams MH Handler CE Akram R Smith CJ Das C Smee J. Role of N-terminal brain natriuretic peptide (N-TproBNP) in scleroderma-associated pulmonary arterial hypertension. Eur Heart J (2006) 27:1485–94. doi: 10.1093/eurheartj/ehi891 16682379

[15] Leuchte HH El Nounou M Tuerpe JC Hartmann B Baumgartner RA Vogeser M. N-terminal pro-brain natriuretic peptide and renal insufficiency as predictors of mortality in pulmonary hypertension. Chest (2007) 131:402–9. doi: 10.1378/chest.06-1758 17296640

[16] Taichman DB McGoon MD Harhay MO Archer-Chicko C Sager JS Murugappan M. Wide variation in clinicians' assessment of New York Heart Association/World Health Organization functional class in patients with pulmonary arterial hypertension. Mayo Clin Proc (2009) 84:586–92. doi: 10.4065/84.7.586 PMC2704130 19567712

[17] Russo MP Fosser SNM Elizondo CM Giunta DH Fuentes NA Grande-Ratti MF. In-Hospital mortality and glycemic control in patients with hospital hyperglycemia. Rev Diabetes Stud (2021) 17:50–6. doi: 10.1900/RDS.2021.17.50 PMC9380085 34852895

[18] Montori VM Bistrian BR McMahon MM. Hyperglycemia in acutely ill patients. JAMA (2002) 288:2167–9. doi: 10.1001/jama.288.17.2167 12413377

[19] Corstjens AM van der Horst IC Zijlstra JG Groeneveld AB Zijlstra F Tulleken JE. Hyperglycaemia in critically ill patients: marker or mediator of mortality? Crit Care (2006) 10:216. doi: 10.1186/cc4957 16834760 PMC1550943

[20] Kitabchi AE Freire AX Umpierrez GE. Evidence for strict inpatient blood glucose control: time to revise glycemic goals in hospitalized patients. Metabolism (2008) 57:116–20. doi: 10.1016/j.metabol.2007.08.014 18078868

[21] Roberts G Sires J Chen A Thynne T Sullivan C Quinn S. A comparison of the stress hyperglycemia ratio, glycemic gap, and glucose to assess the impact of stress-induced hyperglycemia on ischemic stroke outcome. J Diabetes (2021) 13:1034–42. doi: 10.1111/1753-0407.13223 34536055

[22] Krinsley JS. Association between hyperglycemia and increased hospital mortality in a heterogeneous population of critically ill patients. Mayo Clin Proc (2003) 78:1471–8. doi: 10.4065/78.12.1471 14661676

[23] Yendamuri S Fulda GJ Tinkoff GH. Admission hyperglycemia as a prognostic indicator in trauma. J Trauma (2003) 55:33–8. doi: 10.1097/01.TA.0000074434.39928.72 12855878

[24] Umpierrez GE Isaacs SD Bazargan N You X Thaler LM Kitabchi AE. Hyperglycemia: an independent marker of in-hospital mortality in patients with undiagnosed diabetes. J Clin Endocrinol Metab (2002) 87:978–82. doi: 10.1210/jcem.87.3.8341 11889147

[25] Christiansen C Toft P Jorgensen HS Andersen SK Tonnesen E. Hyperglycaemia and mortality in critically ill patients. A prospect study Intensive Care Med (2004) 30:1685–8. doi: 10.1007/s00134-004-2325-2 15148570

[26] Bolk J van der Ploeg T Cornel JH Arnold AE Sepers J Umans VA. Impaired glucose metabolism predicts mortality after a myocardial infarction. Int J Cardiol (2001) 79:207–14. doi: 10.1016/S0167-5273(01)00422-3 11461743

[27] Su YW Hsu CY Guo YW Chen HS. Usefulness of the plasma glucose concentration-to-HbA(1c) ratio in predicting clinical outcomes during acute illness with extreme hyperglycaemia. Diabetes Metab (2017) 43:40–7. doi: 10.1016/j.diabet.2016.07.036 27663631

[28] Kesavadev J Misra A Saboo B Aravind SR Hussain A Czupryniak L. Blood glucose levels should be considered as a new vital sign indicative of prognosis during hospitalization. Diabetes Metab Syndr (2021) 15:221–7. doi: 10.1016/j.dsx.2020.12.032 PMC8049470 33450531

[29] McGrade P Yang S Nugent K. The association between admission glucose levels and outcomes in adults admitted to a tertiary care hospital. J Community Hosp Intern Med Perspect (2019) 9:195–202. doi: 10.1080/20009666.2019.1611318 31258857 PMC6586082

[30] Li L Chen Q Chen Q Wu R Wang S Yao C. Association between blood glucose within 24 hours after intensive care unit admission and prognosis: A retrospective cohort study. Diabetes Metab Syndr Obes (2020) 13:1305–15. doi: 10.2147/DMSO.S250133 PMC7187769 32425565

[31] Soeters MR Soeters PB. The evolutionary benefit of insulin resistance. Clin Nutr (2012) 31:1002–7. doi: 10.1016/j.clnu.2012.05.011 22682085

[32] Ali Abdelhamid Y Kar P Finnis ME Phillips LK Plummer MP Shaw JE. Stress hyperglycaemia in critically ill patients and the subsequent risk of diabetes: a systematic review and meta-analysis. Crit Care (2016) 20:301. doi: 10.1186/s13054-016-1471-6 27677709 PMC5039881

[33] Mifsud S Schembri EL Gruppetta M. Stress-induced hyperglycaemia. Br J Hosp Med (Lond) (2018) 79:634–9. doi: 10.12968/hmed.2018.79.11.634 30418830

[34] McNamara JJ Mills D Aaby GV. Effect of hypertonic glucose on hemorrhagic shock in rabbits. Ann Thorac Surg (1970) 9:116–21. doi: 10.1016/S0003-4975(10)65784-0 5410974

[35] Chernow B Rainey TG Lake CR. Endogenous and exogenous catecholamines in critical care medicine. Crit Care Med (1982) 10:409–16. doi: 10.1097/00003246-198206000-00019 7042207

[36] Scherpereel PA Tavernier B. Perioperative care of diabetic patients. Eur J Anaesthesiol (2001) 18:277–94. doi: 10.1046/j.0265-0215.2001.00876.x 11350470

[37] Marik PE. Critical illness-related corticosteroid insufficiency. Chest (2009) 135:181–93. doi: 10.1378/chest.08-1149 19136406

[38] Chan TM. The permissive effects of glucocorticoid on hepatic gluconeogenesis. Glucagon stimulation of glucose-suppressed gluconeogenesis and inhibition of 6-phosphofructo-1-kinase in hepatocytes from fasted rats. J Biol Chem (1984) 259:7426–32. doi: 10.1016/S0021-9258(17)42808-0 6234302

[39] McMahon M Gerich J Rizza R. Effects of glucocorticoids on carbohydrate metabolism. Diabetes Metab Rev (1988) 4:17–30. doi: 10.1002/dmr.5610040105 3278872

[40] Esposito K Nappo F Marfella R Giugliano G Giugliano F Ciotola M. Inflammatory cytokine concentrations are acutely increased by hyperglycemia in humans: role of oxidative stress. Circulation (2002) 106:2067–72. doi: 10.1161/01.CIR.0000034509.14906.AE 12379575

[41] Lang CH Dobrescu C Bagby GJ. Tumor necrosis factor impairs insulin action on peripheral glucose disposal and hepatic glucose output. Endocrinology (1992) 130:43–52. doi: 10.1210/endo.130.1.1727716 1727716

[42] Stentz FB Umpierrez GE Cuervo R Kitabchi AE. Proinflammatory cytokines, markers of cardiovascular risks, oxidative stress, and lipid peroxidation in patients with hyperglycemic crises. Diabetes (2004) 53:2079–86. doi: 10.2337/diabetes.53.8.2079 15277389

[43] Abernethy AD Stackhouse K Hart S Devendra G Bashore TM Dweik R. Impact of diabetes in patients with pulmonary hypertension. Pulm Circ (2015) 5:117–23. doi: 10.1086/679705 PMC4405710 25992276

[44] Benson L Brittain EL Pugh ME Austin ED Fox K Wheeler L. Impact of diabetes on survival and right ventricular compensation in pulmonary arterial hypertension. Pulm Circ (2014) 4:311–8. doi: 10.1086/675994 PMC4070784 25006450

[45] Ling Y Johnson MK Kiely DG Condliffe R Elliot CA Gibbs JS. Changing demographics, epidemiology, and survival of incident pulmonary arterial hypertension: results from the pulmonary hypertension registry of the United Kingdom and Ireland. Am J Respir Crit Care Med (2012) 186:790–6. doi: 10.1164/rccm.201203-0383OC 22798320

[46] Alamri A Burzangi AS Coats P Watson DG. Untargeted metabolic profiling cell-based approach of pulmonary artery smooth muscle cells in response to high glucose and the effect of the antioxidant vitamins D and E. Metabolites (2018) 8(4):87. doi: 10.3390/metabo8040087 30513640 PMC6316736

[47] Shen H Wang S Zhang C Gao W Cui X Zhang Q. Association of hyperglycemia ratio and ventricular arrhythmia in critically ill patients admitted to the intensive care unit. BMC Cardiovasc Disord (2023) 23:215. doi: 10.1186/s12872-023-03296-7 37118670 PMC10148444

[48] Wallace DC. Diseases of the mitochondrial DNA. Annu Rev Biochem (1992) 61:1175–212. doi: 10.1146/annurev.bi.61.070192.005523 1497308

[49] Wang L Zhou Z Tian X Wang H Yang D Hao Y. Impact of relative blood glucose changes on mortality risk of patient with acute ischemic stroke and treated with mechanical thrombectomy. J Stroke Cerebrovasc Dis (2019) 28:213–9. doi: 10.1016/j.jstrokecerebrovasdis.2018.09.036 30539756

